# Neural substrates of cognitive control under the belief of getting neurofeedback training

**DOI:** 10.3389/fnhum.2013.00914

**Published:** 2013-12-26

**Authors:** Manuel Ninaus, Silvia E. Kober, Matthias Witte, Karl Koschutnig, Matthias Stangl, Christa Neuper, Guilherme Wood

**Affiliations:** ^1^Department of Psychology, University of GrazGraz, Austria; ^2^Aging and Cognition Research Group, German Center for Neurodegenerative Diseases (DZNE)Magdeburg, Germany; ^3^Laboratory of Brain-Computer Interfaces, Institute for Knowledge Discovery, University of Technology GrazGraz, Austria

**Keywords:** cognitive control, anterior insula, neurofeedback, fMRI, self-awareness

## Abstract

Learning to modulate one's own brain activity is the fundament of neurofeedback (NF) applications. Besides the neural networks directly involved in the generation and modulation of the neurophysiological parameter being specifically trained, more general determinants of NF efficacy such as self-referential processes and cognitive control have been frequently disregarded. Nonetheless, deeper insight into these cognitive mechanisms and their neuronal underpinnings sheds light on various open NF related questions concerning individual differences, brain-computer interface (BCI) illiteracy as well as a more general model of NF learning. In this context, we investigated the neuronal substrate of these more general regulatory mechanisms that are engaged when participants believe that they are receiving NF. Twenty healthy participants (40–63 years, 10 female) performed a sham NF paradigm during fMRI scanning. All participants were novices to NF-experiments and were instructed to voluntarily modulate their own brain activity based on a visual display of moving color bars. However, the bar depicted a recording and not the actual brain activity of participants. Reports collected at the end of the experiment indicate that participants were unaware of the sham feedback. In comparison to a passive watching condition, bilateral insula, anterior cingulate cortex and supplementary motor and dorsomedial and lateral prefrontal areas were activated when participants actively tried to control the bar. In contrast, when merely watching moving bars, increased activation in the left angular gyrus was observed. These results show that the intention to control a moving bar is sufficient to engage a broad frontoparietal and cingulo-opercular network involved in cognitive control. The results of the present study indicate that tasks such as those generally employed in NF training recruit the neuronal correlates of cognitive control even when only sham NF is presented.

## Introduction

Neurofeedback (NF) is a kind of biofeedback with the primary goal of helping the user to gain control over specific predefined aspects of his/her brain activity. In typical NF protocols, brain activity is depicted and fed back in real-time to the users. Real-time feedback allows for rewarding desirable patterns of brain activity with visual, auditory or even tactile stimulation and consequently teaching how to control brain activity (Coben and Evans, 2010). NF is employed quite frequently as a treatment for several clinical disorders such as attention and hyperactivity disorders, depression, autism etc. (Lévesque et al., 2006; Angelakis et al., 2007; Coben and Padolsky, 2007; Gevensleben et al., 2009; Niv, 2013). Besides that, NF has become especially interesting for training and improving different cognitive abilities such as working memory and attention (Egner and Gruzelier, 2001, 2004; Vernon et al., 2003; Vernon, 2005). Even sport (Landers et al., 1991) and artistic performances (Gruzelier et al., 2010) are one of the main areas for using NF to enhance performance (Vernon, 2005).

There are several neurophysiological methods to provide NF, such as real-time fMRI, MEG and functional near infrared spectroscopy or even invasive methods such as electrocorticograms (e.g., Kober et al., 2013b; Ruiz et al., 2013b; Sulzer et al., 2013b). For instance, the application of real-time fMRI is growing and evolving rapidly (for reviews see e.g., Ruiz et al., 2013a; Sulzer et al., 2013a). However, especially regarding the feedback speed and the depicted brain signal, EEG NF and real-time fMRI differs significantly (e.g., Sulzer et al., 2013a).

Regardless of which method is employed to provide NF, several aspects of NF learning are constant. An increased degree of attention to the inner state to be learned by NF, a reduction of motor activity and artifacts, general relaxation, concentration, etc., are necessary to learn from NF. Moreover, the usually vague verbal strategies given to participants keep a high degree of similarity across different NF protocols: participants are generally asked to relax and concentrate on the feedback regardless of the exact neurophysiological parameter being trained (Kropotov, 2009). It is recognized that implicit learning mechanisms play an important part in regulating brain activity (e.g., Birbaumer et al., 2013). Birbaumer and colleagues propose that brain-self-regulation is not necessarily an explicit and conscious process and is very similar to skill learning. For instance, real-time fMRI studies show that not only pure operant learning (e.g., Shibata et al., 2012), but also mental imagery and explicit strategies (Kober et al., 2013a,b; Ruiz et al., 2013b) empower participants to self-regulate different brain regions (for reviews see e.g., Birbaumer et al., 2013; Sulzer et al., 2013a). The interplay between skill learning and conscious processes in NF is still poorly understood and the meaning of control over brain activity behind these two processes differs in a very fundamental way. While skill learning describes how specific networks in the brain are modulated by NF on their own activity, explicit processes have a much more general function. Explicit processing and cognitive control may help to calibrate the activation in the rest of the brain through executive processes with the view of not disturbing the networks learning from NF. Hitherto, there is no framework integrating into a unified neurocognitive model this dissimilar collection of aspects of NF learning. In face of the large interest in NF and its good reputation (AAP, 2013), it is necessary that more fundamental principles of NF learning as well as its more general neuronal correlates can be distinguished (Kropotov, 2009).

It is known that explicit learning mechanisms and top-down processes can have a substantial impact on implicit or bottom-up mechanisms (Shallice and Cooper, 2011), however, research on NF is scarce regarding the influence of top-down processing and cognitive control on NF learning. Therefore, in the present study, we aimed to go a step forward toward the understanding of explicit control mechanisms related to NF learning. Particularly, the neural correlates of self-referential processes such as the attention to inner states as well as cognitive control during a NF-like task will be investigated.

A closer inspection of the structure of NF setup reveals that this skill depends directly on focusing attention on internal states. This implies the ability to reduce the attention to external events and concentrate over a determined period of time on internal states. Other cognitive states such as meditation (e.g., Lazar et al., 2005; Farb et al., 2007) and mind wandering (e.g., Mason et al., 2007; Vanhaudenhuyse et al., 2011) are paradigmatic regarding the focusing of attention to internal states. Several studies show that the anterior part of the insula plays a part in attention to and the awareness of internal cues (Barrett et al., 2004; Critchley et al., 2004; Pollatos et al., 2007). Accordingly, learning from NF is most likely dependent on a strong subjective momentary interoceptive sensory process of the participant.

Another central aspect of NF is the perception of control over brain activity. When participants believe to have control over a NF protocol, they refer to the feeling of causing the action, meaning that the participants believe they are able to control the NF in an appropriate way. In the literature this phenomenon is called agency (Gallagher, 2000). Agency plays an important role in self-consciousness and tells us whether an action is caused by ourselves or other entities (Gallagher, 2000; Newen and Vogeley, 2003). Recently, the anterior insula has been identified as a central hub for self-agency (Sperduti et al., 2011). As mentioned above, the primary goal of NF is to gain control over specific predefined aspects of the brain activity. Due to the immediate feedback in conventional NF protocols, self-agency and agency in general, respectively, could play an important part in NF trainings.

Self-referential processes such as agency and self-awareness play an important role in gaining control over brain activity. Therefore, brain regions supporting interoceptive attention such as cingulate cortex, supplementary motor area, dorsomedial and lateral prefrontal areas and especially the insula, as a hub for self-referential processes, can be relevant for NF training because they are involved in the regulation of neuronal activity in many other regions of the brain, which may both favor or hinder NF learning. Evidence suggests that different forms of control (e.g., overcoming cognitive interference, adjusting performance after making an error, inhibiting a prepotent response, regulating one's drug craving, etc.; for reviews see e.g., Miller, 2000; Miller and Cohen, 2001; Kana et al., 2007; Dosenbach et al., 2008; Garavan et al., 2013; Power and Petersen, 2013) engage at least partly overlapping brain networks. So it is plausible that the subjective feeling emerging when one is engaged in a task which demands learning from feedback will be accompanied by the activation in brain areas involved in cognitive control.

To our knowledge, no studies have examined hitherto the neuronal correlates of cognitive control under the belief of training neurofeedback. In this study we examine the cognitive mechanisms and neuronal underpinnings of perceived levels of control over NF applications. In this context, our main goal was to investigate the brain activations observed when participants have the intention to control a moving bar, a task such as those generally employed in NF training. Since we are interested in the neural networks associated with the belief of control in a NF situation and not in the capacity to learn from NF, no real NF protocol was employed in the present study but rather a condition of realistic but fake feedback. In most of the published NF studies, participants do not have a very well specified representation of how to modulate or influence the NF paradigm (e.g., Vernon et al., 2003; Gruzelier et al., 2010; Weber et al., 2011; van Boxtel et al., 2012). For this reason we expected to elicit genuine control beliefs regarding NF regulation in our participants. Sham NF was presented in form of moving bars, which according to instructions were representing participants' brain activity in a very realistic way. To examine neural correlates of participants' attempts to control the NF interface, functional magnetic resonance imaging (fMRI) was employed. Every participant received exactly the same visual feedback. With this methodological approach, we were able to examine the neurophysiological response on the participants' attempt to interact with a NF-like paradigm, independent of individual success rates or better and worse performers in general. Additionally, we were interested in how much control beliefs affect neurophysiological and behavioral correlates of NF learning. For that we have used questionnaires assessing control beliefs while dealing with technology in general.

## Materials and methods

### Participants

Twenty volunteers [10 male, 10 female, age range 40–63 years; mean age = 46.4 years; standard deviation (*SD*) = 5.14] participated in the experiment after giving informed consent. All participants were naïve to the purpose of the study, had normal or corrected to normal vision and presented no history of major medical illness, neurological or psychiatric disorder, or substance abuse. At the end of the experiment, participants were informed that the feedback presented in fMRI was not related to their brain activation but was a mere recording. The experimental protocol was approved by the ethics committee of the University of Graz.

### Tasks

#### Experimental task

**Figure 1:**
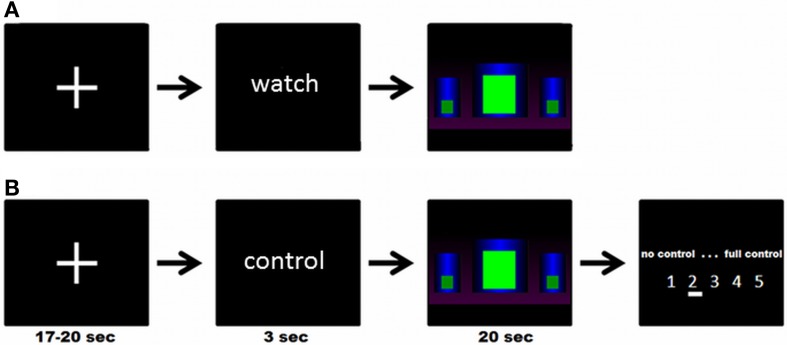
**(A)** Example of a complete trial of the “watch moving/static bars” condition; **(B)** Example of a complete trial of the “get control” condition; participants were instructed to try to control the movements of the bars during functional MRI measurement.

There were three different block designed modeled conditions: in the experimental condition (“get control”), participants were instructed to get control over the movement of the bar by using their brain activity. In the two control conditions, participants were instructed to watch passively the bars. In one control condition (“watch moving bars”) the bars were moving like in the “get control” condition and in the other control condition the bars were static (“watch static bars”). Each condition was repeated five times. At the beginning of each trial a cross-hair has been presented for about 18.5 s (jittered from 17 to 20 s). Following this, participants received the visual cues “control” or “watch” for 3 s to get ready for the three different conditions. No action was required in this part of the trial. In the next part of the trial the participants were able to see three different bars. In the “get control”- condition, participants were instructed to try to control the bars during a time interval of 20 s and, at the end of each interval, to rate their perceived success controlling the bars afterwards on a 5-point rating scale (no control—full control). In the “watch moving bars” and the “watch static bars” conditions, participants were instructed to watch passively the screen for 20 s. The timing and the visual appearance of a trial is also shown in Figure 1.

The experimental task was conceived to resemble as closely as possible a genuine NF training session. At the beginning of the session, participants were told that they would take part in a training study and that they should use the visual feedback given by a moving bar displayed inside the fMRI scanner to voluntarily modulate their own brain activity according to the feedback. On a feedback screen participants were able to see three different bars (see Figure 1). They were instructed to increase the middle bar and decrease the left and right bar. The participants have not received an explicit strategy on how to gain control over the feedback and therefore our approach is comparable with conventional EEG NF instructions (e.g., see Kober et al., 2013a; Witte et al., 2013). The instructions were the same as those our lab uses in the training of SMR frequency in EEG (Witte et al., 2013). Participants were instructed to relax and keep concentrated for the time period of experimental blocks and to use the pauses between them to recover. More specific strategies about how participants were supposed to comply with the instructions were not provided. The visual display was a recording of different sessions of SMR NF training with EEG. Participants were not informed about the exact meaning of the different bars but only that it was important for them to succeed that the central bar should be kept as high as possible and the two lateral bars as low as possible. The animation of the bars was updated 20 times per second and produced by sampling authentic EEG signal of persons undergoing NF training. Therefore, the movements were naturalistic and representative of a typical NF session in both “watch moving bars” and “get control” conditions. Data selected for the production of stimuli were filtered to eliminate movement and eye artifacts. Search for artifacts was conducted by two independent and experienced EEG analysts. This signal was smoothed with a moving average of 1 s so that jumps in signal were avoided. At the moment of fMRI data acquisition, participants were unaware that they got sham feedback. With this methodological approach we were able to examine the neurophysiological response on the participants' efforts to gain control over their own brain activity.

#### Locus of control for technology

After the fMRI session, participants were asked to fill out several questionnaires. The “locus of control for technology”-questionnaire (KUT; Beier, 2004) was used to assess control beliefs while dealing with technology. The KUT-questionnaire asks the participants to rate, on a 5-point Likert-scale, their handling of technology on 8 items (range of score: 8–40). More precisely the KUT-questionnaire assesses the specific interaction with technical environments of users (e.g., “*I really enjoy finding a solution for technological problems*”; “*Most of the technological problems that I have to face can be solved by myself*.”). The questionnaire is available in German and has a high reliability (α = 0.89; Beier, 1999). Recently, Witte et al. (2013) showed that control beliefs, assessed with the KUT-questionnaire can predict the ability to control a NF. Furthermore, Burde and Blankertz (2006) demonstrated in a BCI-study that the higher the score in the KUT questionnaire was the better was the BCI performance.

#### Rumination scales

Rumination, a method of coping with negative mood that involves self-focused attention as well as self-reflection (Morrow and Nolen-Hoeksema, 1990; Lyubomirsky and Nolen-Hoeksema, 1993), was assessed with the short version of the Ruminative Response Scale (RRS; Treynor et al., 2003). The RRS is a self-reported measure of rumination consisting of 10 items, and it relates to different components of rumination: reflective pondering and brooding. The first, reflective pondering is an adaptive type of rumination that describes the degree of engagement in cognitive problem solving recruited to improve mood. Depressive brooding is a maladaptive type of rumination which reflects the focus on the meaning and symptoms of distress (De Lissnyder et al., 2012).

### MRI data acquisition

Neuroimaging data were acquired with a 3.0 Tesla Siemens Skyra MRI scanner at the MRI-Lab Graz (Austria) using a 32 channel head coil and parallel imaging with an iPAT acceleration factor of 2. Functional images were acquired using a T2^*^ weighted gradient-echo pulse imaging sequence (TR = 920 ms; TE = 30 ms, flip angle = 72°; 64 × 64 matrix; voxel dimensions = 4 × 4 × 4 mm), providing whole brain coverage in 23 slices.

The participants were positioned comfortably in a supine orientation with their head located in the head coil. Foam padding was used around the head to minimize head movements. Participants wore earplugs to reduce discomfort due to scanner noise. Participants viewed the experimental protocol on a screen, via a mirror attached to the head coil. Behavioral measures (ratings) were collected via a MR compatible response box. Participants were required to press the buttons under their index finger and ring finger to navigate through the rating possibilities and to confirm their decision, participants had to press the button under their middle finger.

### fMRI data analysis

Functional data were preprocessed and analyzed using SPM8 (http://www.fil.ion.ucl.ac.uk/spm/). For the preprocessing of the functional MRI images we used the “MoCo”-series provided by Siemens (Siemens, www.medical.siemens.com), which were corrected retrospectively for intrascan movement. The fMRI-data were then realigned using the first scan as a reference to which all subsequent scans are realigned. Slice time correction was performed. A mean image created from the realigned volumes was spatially normalized to the Montreal Neurological Institute (MNI) EPI brain template (SPM8). The derived spatial transformation was then applied to the realigned and slice time corrected T2^*^ volumes, which were finally spatially smoothed to facilitate group level statistics with a Gaussian kernel of 8-mm FWHM. Statistical models were constructed on the basis of the general linear model implemented in SPM8: all conditions fixation point, cue, “watch static bars,” “watch moving bars,” “get control,” and rating were modeled as block design conditions in a single design matrix. Contrasts were estimated for each participant individually in a first level analysis and statistically tested in second level analyses. Of primary interest for this study were the contrasts of “get control” > “watch moving bars” and “watch moving bars” > “get control.” Also reported were the contrasts “watch moving bars” > “watch static bars” and “watch moving bars” < “watch static bars.” Whole brain analysis results are reported at a threshold of *p* < 0.001 uncorrected and *p* < 0.05 corrected for multiple comparisons on cluster-level [false discovery rate (FDR)] with a minimum cluster size of 10 voxels. All reported coordinates are reported in MNI space.

## Results

### Behavioral results

Participants had to rate their success controlling the bars on a 5-point rating scale (1 = no control to 5 = full control). The mean rating of success of all participants was 3 (3 = medium control; mean rating = 2.69; *SD* = 0.66; Range: 1–4; Table 1). None of the participants reported that they were aware of the sham feedback during a debrief session.

**Table 1:** **Correlation between performance rating, control beliefs, and rumination**.

**Results correlations**							**Means**	***SD***
		**Rating**	**KUT**	**RRS**				
					Reflection	Brooding		
Rating		1.00					2.69	0.66
KUT		−0.46^*^	1.00				31.40	5.61
RRS		0.01	0.02	1.00			23.20	2.84
	Reflection	−0.16	0.19	0.55^*^	1.00		12.85	2.98
	Brooding	0.19	−0.18	0.43	−0.51^*^	1.00	10.35	2.76

KUT, “locus of control while dealing with technology”-questionnaire; RRS, Ruminative Response Scale;

* *p < 0.05*.

The average score on the KUT-questionnaire of the participants was 31.4 (*SD* = 5.61; Minimum = 20, Maximum = 38). On the RRS questionnaire, participants had an average score of 12.85 (*SD* = 2.98) for the component reflection and 10.35 (*SD* = 2.76) for the component brooding (Table 1).

To address the relation between performance rating, control beliefs while dealing with technology and rumination, Pearson correlations were calculated (Table 1). A significant negative correlation was found between the performance rating and KUT-scores.

### Neuroimaging results

**Figure 2:**
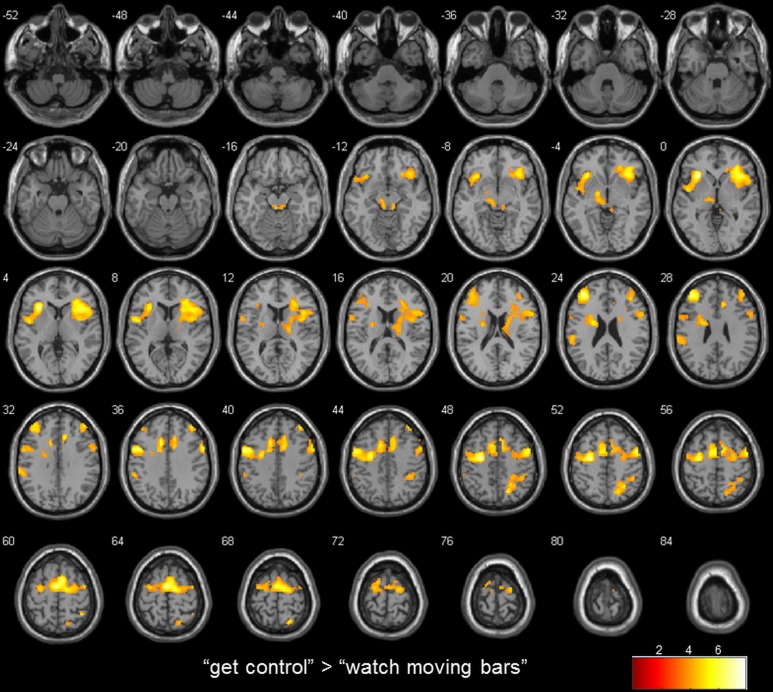
**transversal slices (4 mm spacing); t-score map for “get control” “watch moving bars”; *p* < 0.001 uncorrected on voxel-level, *p* < 0.05 corrected for multiple comparisons on cluster-level [false discovery rate (FDR)]; minimum cluster size 10 voxels**.

Whole brain analysis results are reported at a threshold of *p* < 0.001 uncorrected in pictures and *p* < 0.05 corrected for multiple comparisons on cluster-level [false discovery rate (FDR)] with a minimum cluster size of 10 voxels in tables. All reported coordinates are reported in MNI space (Table 2; Figure 2).

**Table 2:** **Brain regions preferentially activated when attempting to get control over moving color bars compared to when passively watching the moving color bars**.

	**Brodmann areas**	**Voxels**	**Peak**			***T*-value**
			***x***	***y***	***z***	
**“get control” > “watch moving bars”**						
*L insula*	6, 13, 32, 9, 47, 44, 24, 46, 10, 45, 22, 8, 4, 38,	3748	−30	23	1	7.87
*- R insula*						
*- L precentral gyrus*						
*- dorsomedial and lateral prefrontal area*						
*- bilateral supplementary motor area*						
*- L anterior cingulate gyrus*						
*- R pars opercularis*						
*R sup. parietal lobe*	7	229	18	−64	49	5.60
*R middle frontal gyrus*	9, 10, 46	110	39	41	40	4.07
*L thalamus*		149	−15	−10	−2	4.98
*L supramarginal gyrus*	40	114	−57	−37	34	4.67
**“watch moving bars” > “get control”**						
*L angular gyrus*	39	90	−48	−70	28	7.91

*Reported coordinates in MNI space; L, left; R, right; p < 0.001 uncorrected on voxel-level, p < 0.05 corrected for multiple comparisons on cluster-level [false discovery rate (FDR)]; minimum cluster size 10 voxels*.

To examine the extent to which self-referential processes are relevant for the perceived level of control over a NF-like task, we contrasted the conditions “get control” and “watch moving bars.” Importantly, this contrast revealed several highly significant activation clusters. A widespread activation has been identified in frontal areas with its peak in the left anterior part of the insula. Additionally, a large cluster of activation including the right insula dorsomedial and lateral prefrontal and bilateral supplementary motor area as well as the anterior part of the cingulate gyrus. Furthermore, this comparison also revealed significant cluster activation in the right superior parietal lobe, right middle frontal gyrus, left supramarginal gyrus and left thalamus. The corresponding Brodmann areas of these significant activation clusters are listed in Table 2. In contrast during the simply watching trials, compared to attempt of controlling the bars, significant activation has been identified only in left angular gyrus (Table 2; Figure 2).

**Figure 3:**
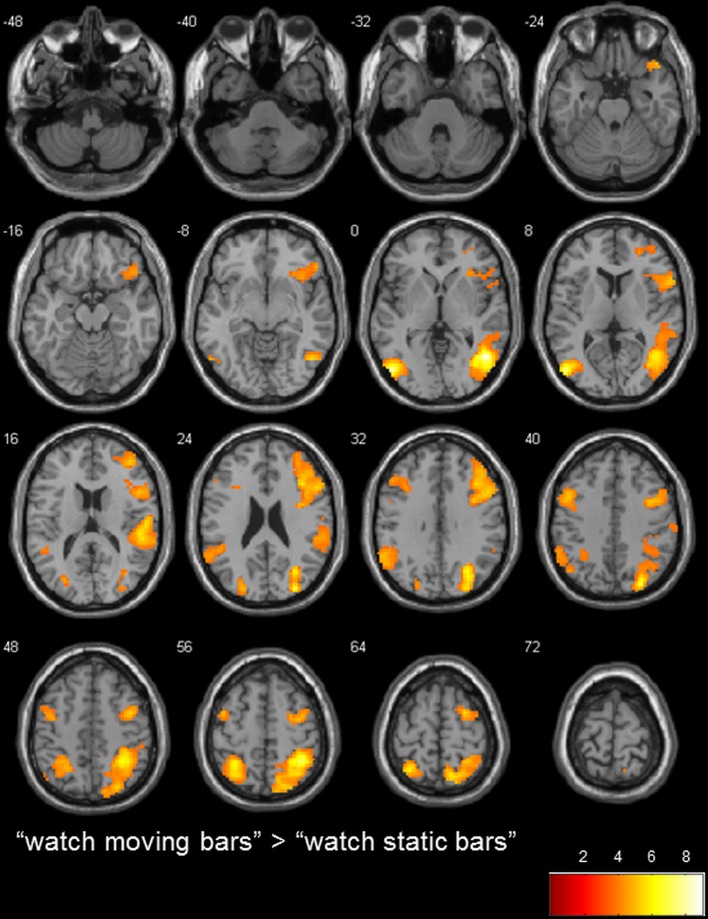
**transversal slices (8 mm spacing); t-score map for “watch moving bars” > “watch static bars”; *p* < 0.001 uncorrected on voxel-level, *p* < 0.05 corrected for multiple comparisons on cluster-level (false discovery rate [FDR]); minimum cluster size 10 voxels**.

To determine the general level of activation when participants observed a moving bar, we used the contrasts of “watch moving bars” > “watch static bars” and “watch static bars” > “watch moving bars.” A broad network of activation covering the right and left temporo-parietal and inferior frontal areas was observed after subtracting the activity of “watch static bar” from “watch moving bars” (Table 3, Figure 3).

**Table 3:** **Brain regions preferentially activated when observing moving color bars compared to static color bars (low level control condition)**.

	**Brodmann areas**	**Voxels**	**Peak**			***T*-value**
			***x***	***y***	***z***	
**“watch moving bars” > “watch static bars”**						
*R temporal cortex, inferior and superior parietal cortex, fusiform gyrus, posterior insula*	7, 40, 19, 37, 41, 39, 13, 5, 42, 22	2236	48	−61	1	8.99
*L middle occipital gyrus*	37, 19, 39	328	−51	−76	1	7.92
*L superior parietal lobule*	7, 40	364	−30	−52	55	6.05
*R inferior frontal gyrus*	9, 6, 45, 10, 47, 46, 44, 8	1439	39	23	28	5.89
*L supramarginal gyrus*	40, 39	280	−60	−52	28	5.15
*L precentral gyrus*	6, 9, 8	264	−42	−1	52	5.10
**“watch static bars” > “watch moving bars”**						
*No significant activation cluster*						

*Reported coordinates in MNI space; L, left; R, right; p < 0.001 uncorrected on voxel-level, p < 0.05 corrected for multiple comparisons on cluster-level [false discovery rate (FDR)]; minimum cluster size 10 voxels*.

## Discussion

The aim of the present study was to investigate the neural basis of participants' subjective experience when they believe to have control over an external device and use visual feedback. To be able to disentangle the effect of subjective experience from learning from NF, we hold learning rates constant by using sham feedback. Participants were instructed to try to get control over a moving bar presented on the screen in the same way as in a real training session with NF. A large network involving the anterior insula, bilaterally, right operculum, ventral, dorsomedial and -lateral prefrontal cortex, inferior parietal lobule, supplementary motor area and anterior cingulate cortices was more activated when participants actively engaged in the task to move the bar than when they passively watched the bar movements. Moreover, the perceived ability to control the bar during the fMRI measurement correlated negatively with control believes toward electronic devices. In the following, these results will be discussed in more detail.

### Performance rating

Debrief performed after the fMRI measurement revealed that no participant questioned the veracity of the feedback obtained in the “watch moving bars” and “get control” conditions. The rating provided by participants after finishing each run of the “get control” condition reveals that participants were actively engaged in the task of trying to get control over the movements of the bar presented on the screen. Average scores in the rating were close to the absolute arithmetical mean of the rating interval employed. This can be interpreted as evidence that most participants perceived their ability to control the bar as superior to the chance level. Interestingly, these ratings also were negatively correlated with the control believes toward electronic devices as reported in the KUT scale. These results show that the higher the perceived ability to control electronic devices (i.e., operate a computer or a mobile phone), the lower the perceived ability to control the moving bar during the experiment. Those participants reporting higher control believes toward electronics correctly assessed the levels of control over the bar, which was definitely not under their control, as lower. This suggests that the higher the levels of perceived control reported by participants in general, the higher were also the expectations regarding the capacity to control one's own brain activity and consequently to learn from NF. In participants with high expectations regarding their performance controlling the bar the frustration as expressed by the low ratings was more pronounced. In general, the perception about the general ability to control electronic devices seems to be a determinant of the perceived success of NF learning (see also Witte et al., 2013).

In the present study, most participants reported moderate levels of perceived control over bar movements when in reality they had absolutely no control over it. One could argue that these results are due to the complete absence of valid feedback to participants. However, results from double-blind NF studies (e.g., Witte et al., 2013) reveal that a similar pattern is observed also when real NF is applied. In these studies, most participants receiving either real or sham NF are not able to guess to which group they were assigned even after many successful training sessions. This shows that even when NF is applied, the perception of control is not accurate in NF tasks. To the contrary, perception of control in NF tasks is inaccurate most of the time during training because thresholds are adjusted on a regular basis and individual performance is very variable across sessions. Therefore, it is of primordial interest to detect the brain networks related to believed control. In the following section these results will be discussed in more detail.

### The neuronal topography of cognitive control in a neurofeedback-like paradigm

To our knowledge, this is the first study which examines the neural correlates of executive control in a NF-like paradigm using fMRI. In comparison to the control condition “watch static bars,” stronger activation in a broad network of regions distributed over the right temporal cortex, inferior and superior parietal cortex, fusiform gyrus, posterior insula, left middle occipital gyrus, left superior parietal lobule, right inferior frontal gyrus, left supramarginal gyrus and left precentral gyrus was observed in the condition “watch moving bars.” The activations observed in occipital, temporal and parietal cortex reflect probably the processing of bar movements (Burr and Morrone, 2012), while the activations observed in the inferior frontal gyrus, bilaterally, reflect probably an increase of attention to inner states (Craig, 2009), since in this condition participants were told that they were seeing their own brain activity represented by the moving bar.

To investigate the specific correlates of trying to get control over the bar movements, the conditions “get control” and “watch moving bars” were compared directly. This contrast allows for the interpretation of those neural correlates of cognitive control recruited in situations comparable to NF training uncontaminated by the processing of movement, since the bars moved according to the same principles and in comparable amount in both conditions.

The neuroanatomical structure showing the strongest activation in the contrast “get control” > “watch the bar” was the bilateral anterior insula, which is mainly responsible for driving attention to specific inner states. Our results suggest that the insula is providing the circuitry related to cognitive control with a reference against which to compare the incoming information from visual feedback. Based on the match or mismatch between these two pieces of information, the structures related to cognitive control continuously calibrate cognitive activation in an effort to improve the match between feedback and inner states (Dosenbach et al., 2007). Since feedback was not genuine, the mismatch between inner and external states was constant and cognitive control had to be continuously applied in the “get control” condition. NF learning requires the participants to focus on their own physiological states but the information of their inner state is provided by an external medium (e.g., visual display, auditory feedback etc.). Hence, this process is facilitated by the engagement of cognitive resources dedicated to self-referential processes to integrate this external information into the self.

In other words, the participation of the anterior insula in the present study may be understood as a central hub, which compares and integrates the external information provided by the feedback display with internal information regarding brain activity (Craig, 2009). The feedback, when effective, would enable participants to learn the mental representation of the interrelationship between oneself and the feedback bars in the immediate moment. Therefore, NF could be seen as an embodied tool, meaning that the users embody the provided feedback into their self, in a comparable way as in the BCI literature (e.g., O'Hara et al., 2011; Heersmink, 2013).

Along with the importance of the insula in integrating information provided by the feedback into self-related processes, the participation of anterior cingulate and dorsolateral prefrontal cortex during the “get control” shed further light onto the neural bases of the process of NF learning. NF requires participants to compare the actual state with the desired state of the feedback to be able to learn how to get control over the NF paradigm. The anterior cingulate cortex is known to be associated to detect discrepancies between an actual and a desired state (Carter et al., 2000; Kerns et al., 2004), self-reflection (Herwig et al., 2012) and to tuning attentional processes (Bishop et al., 2004) with direct connections to thalamus, insular cortex, amygdala, parietal and prefrontal areas (Goldman-Rakic, 1988). Describing these functions of the anterior cingulate cortex highlights the importance of this region for NF regardless of the content of specific NF training programs.

### Attention-network under the belief of getting neurofeedback training

Besides the increase in bilateral insula activation during the “get control” condition, activation in the right pars opercularis of the inferior frontal gyrus and right middle frontal gyrus was observed. In several studies, those two regions are associated with stimulus-driven attention and the maintaining of attention (e.g., Corbetta and Shulman, 2002; Weissman et al., 2006). Furthermore, the whole brain analysis revealed activation in several regions associated with cognitive control, such as dorsomedial and lateral prefrontal areas. During the “get control” condition, participants had to sustain their attention toward internal and external sources over a period of time. Along with the identified activation in right pars opercularis, and right middle frontal gyrus and dorsomedial and lateral prefrontal areas we have found activation in a brain structure closely associated with the left thalamus. Those brain areas are critical for processing internal states (Miller and Cohen, 2001) and therefore could play a critical role in acquiring control over a NF paradigm. Further evidence that the participants were highly engaged in acquiring control over the bars and shifted their attention toward the sham NF is the significant bilateral activation in the supplementary motor area, especially in context with the significant activation in anterior cingulate cortex and thalamus during the “get control” condition. Those regions provide signals that support the brain's moment-to-moment information processing and form a centralized control system (Dosenbach et al., 2007). Regarding our paradigm, where participants had to constantly integrate the provided information from the moving bars, the identified regions apparently play a major role in driving the regulation of inner states with the aim of regulating the movements of the bar. However, we cannot rule out that the bilateral activation in the supplementary motor area emerged due to anticipation of motor response during the “get control” condition since participants were asked to rate their performance after the feedback in the “get control” condition.

### Left angular gyrus

The contrast between “watch moving bars” and “get control” condition revealed only one significant activation cluster with its peak in the ventral part of the left angular gyrus. Seghier (Seghier et al., 2010; Seghier, 2013) proposed that this part of the left angular gyrus is associated with the default mode network. Therefore, left angular gyrus is prominent during rest and when persons are not engaged in external interactions (Buckner et al., 2008) and is interrupted during effortful tasks (Binder et al., 1999; Seghier et al., 2010). Our result implicates that the participants did not try to gain control or interact with feedback during the “watch moving bars” condition, which supports our experimental setup.

However, a meta-analysis about different brain structures related to self- and external-agency conducted by Sperduti and colleagues (Sperduti et al., 2011) revealed that the left angular gyrus is also part of the external agency attribution network. During the “watch moving bars”-condition the participants may have experienced that the displayed moving bars are rather not caused by them than during the “get control” condition.

### Control beliefs may influence neurofeedback learning

As outlined so far, NF training is linked to interoceptive and self-referential processes. In contrast to NF trainings, in most of the brain computer interface studies participants get a quite concrete instruction about how they are able to control the interface or what mental task they should use. The common mental tasks are motor imagery, mental subtraction etc. (e.g., Friedrich et al., 2012) to yield highly distinguishable brain patterns. It has been shown that control beliefs toward technology can be used as a predictor of brain computer interface performance. Burde and Blankertz (2006) showed that the higher the KUT results were the better was the brain computer interface performance. Due to the fact that gaining control over a NF especially in the early stages of a training mostly relies on “trial-and-error” learning and that the participants do not receive a concrete instruction about how to modulate the NF paradigm, it is not surprisingly that the KUT score in the present study negatively correlates with ratings of the participants after a “get control” trial. Participants with a higher KUT score may expect that they are able to deal well with the NF paradigm form the beginning. These participants have the most reasons to be disappointed with their performance and therefore rate their success to control the NF lower than participants with a lower KUT score and lower expectations regarding the outcome (see also Witte et al., 2013). These findings could also imply that participants who are highly convinced handling technology well struggle the most with getting control over a NF. In a recent study, Kober et al. (2013a) showed that some strategies employed by NF participants may lead to a cognitive overload, which prevents NF learning. Due to the repeated attempts by those persons to get control over the NF, they may impede themselves by trying too hard and are therefore not able to direct their attention on the quite subtle internal bodily cues, especially at the early stages of the training.

### Summary

In summary, we used fMRI to identify brain regions associated with believed control in a task similar to NF learning. Because of the use of sham feedback only, the activations observed in the present study are due to differences in believed control and were not affected by specific NF learning processes. The significant activation in the anterior insular and cingulate cortex suggests that participants actively tried to get control over the moving bar in the “get control” condition. Behavioral data additionally showed a negative correlation between the subjective estimates about the amount of control perceived in the “get control” condition and control beliefs.

The present study reveals the neuronal networks related to general regulatory processes associated with NF settings. We assume that especially regions relevant for self-referential processes such as self-awareness and self-agency play an important part in acquiring control over a NF, due to the fact that participants have to focus on their own physiological signals, which requires a big amount of self-referential cognitive load.

### Conflict of interest statement

The authors declare that the research was conducted in the absence of any commercial or financial relationships that could be construed as a potential conflict of interest.
